# Development of a new hazard scoring system in primary neuronal cell cultures for drug-induced acute neuronal toxicity identification in early drug discovery

**DOI:** 10.3389/fphar.2024.1308547

**Published:** 2024-05-30

**Authors:** Mohamed Kreir, Dea Putri, Fetene Tekle, Francesca Pibiri, Constantin d’Ydewalle, Karel Van Ammel, Helena Geys, Ard Teisman, David J. Gallacher, Hua Rong Lu

**Affiliations:** ^1^ Global Toxicology and Safety Pharmacology, Preclinical Sciences and Translational Safety, Janssen Research and Development, Beerse, Belgium; ^2^ Statistics and Decision Sciences, Global Development, Janssen Research and Development, Beerse, Belgium; ^3^ Neuroscience Discovery, Janssen Research and Development, Beerse, Belgium

**Keywords:** neuronal cells, micro-electrode array (MEA), neuronal toxicity, seizures, adverse effect (AE), hazard score system

## Abstract

We investigated drug-induced acute neuronal electrophysiological changes using Micro-Electrode arrays (MEA) to rat primary neuronal cell cultures. Data based on 6-key MEA parameters were analyzed for plate-to-plate vehicle variability, effects of positive and negative controls, as well as data from over 100 reference drugs, mostly known to have pharmacological phenotypic and clinical outcomes. A Least Absolute Shrinkage and Selection Operator (LASSO) regression, coupled with expert evaluation helped to identify the 6-key parameters from many other MEA parameters to evaluate the drug-induced acute neuronal changes. Calculating the statistical tolerance intervals for negative-positive control effects on those 4-key parameters helped us to develop a new weighted hazard scoring system on drug-induced potential central nervous system (CNS) adverse effects (AEs). The weighted total score, integrating the effects of a drug candidate on the identified six-pivotal parameters, simply determines if the testing compound/concentration induces potential CNS AEs. Hereto, it uses four different categories of hazard scores: non-neuroactive, neuroactive, hazard, or high hazard categories. This new scoring system was successfully applied to differentiate the new compounds with or without CNS AEs, and the results were correlated with the outcome of *in vivo* studies in mice for one internal program. Furthermore, the Random Forest classification method was used to obtain the probability that the effect of a compound is either inhibitory or excitatory. In conclusion, this new neuronal scoring system on the cell assay is actively applied in the early de-risking of drug development and reduces the use of animals and associated costs.

## 1 Introduction

In addition to cardiac and liver liability, neuronal toxicity, including seizure liability, represents a major safety concern in drug development, given the potentially life-threatening consequences for patients (Authier et al., 2016). Therefore, an early assessment of seizures within drug discovery is essential to advance promising, and more sustainable, new chemical entities into clinical evaluation. As a result, late-stage attrition caused by seizures could be greatly avoided, reducing the potential risk to participants in clinical studies and the associated costs of getting to this stage. Drug-induced seizures are a common Central Nervous System (CNS)-related issue during drug development and are deemed serious, potentially life-threatening adverse reactions that can result in the withdrawal of drugs from the market (Onakpoya et al., 2016a; Authier et al., 2016; Onakpoya et al., 2016b), prescription control, or the discontinuation of further drug-candidate development at various Research and Development (R&D) stages. *In vivo* models have been widely used to assess seizure liability. However, the general investigation of CNS safety employing the modified Irwin test is often carried out in the latter stages of the drug development process (Irwin, 1968; Gauvin and Zimmermann, 2019; Redfern et al., 2019). In recent years, *in vitro* assays to study neuronal activity have been shown to be useful for early screening of CNS toxicity, including seizures (Kreir et al., 2018a; Bradley et al., 2018; Shafer et al., 2019). Primary cortical neuronal cultures grown on micro-electrode arrays (MEAs) exhibit spontaneous electrical spikes and clusters of spikes (bursts) that are associated with neuronal action potentials and could be used to detect drug-induced phenotypic effects on MEAs (Wheeler et al., 2004). These cultures show synchronous and rhythmic neuronal activity, which is related to synaptogenesis and the equilibrium of excitatory and inhibitory synapses (Muramoto et al., 1993; Maeda et al., 1995; Chiappalone et al., 2006). Cortical networks are responsive to various chemicals (neuronal transmissions), including direct agonists and antagonists of glutamatergic and GABAergic receptors, voltage-gated sodium channels, and glutamatergic and GABAergic channels (Campillos et al., 2008; Shafer et al., 2008; Scelfo et al., 2012; Baskar and Murthy, 2018). Studies using primary cortical neurons demonstrated that measuring the changes of extracellular action potentials in the neuronal network using the MEAs can be used for screening compounds for neurotoxicity hazards (Frega et al., 2012; Lantz et al., 2014; Bradley et al., 2018; Bradley and Strock, 2019). However, the identification of the risk of compounds based on disturbances of neuronal activity *in vitro* remains a challenging task due to the complex pattern and often too many parameters of neuronal activity measured by MEA. Current MEA analysis approaches need the use of raster plots to visualize network changes or individual or multiple-parameter analysis, which is qualitative and difficult to interpret, respectively. While the general pattern of neuronal network changes after drug exposure has been described in previous studies (Kreir et al., 2018a), there is a lack of sufficient methods to quantify the observed effects in a much-simplified manner. Here, we develop a new simplified method to create a hazard scoring system based on six key parameters of MEA recordings, to give a single outcome (scoring) of drug-induced seizure potentials to deselect unwanted compounds in early drug discovery. This outcome is simplified as a neuronal hazard scoring system that serves as an easily interpretable measurement to evaluate the effects of various treatments. We present the characterization and development of this method in this study using a large set of reference drugs, known to have certain CNS pharmacological effects and clinical outcomes. Therefore, it demonstrates the ability to measure the balance of neuronal electrophysiological effects (from both positive and negative effects) on neural network activity and further interrogates the potential of compounds in different acute neuronal hazard scorings.

## 2 Materials and methods

### 2.1 Ethical procedures

All experiments involving the use of animals have been conducted in accordance with the European directive of 2010 (2010/63/EU) on the protection of animals used for scientific purposes and the Belgian Royal Decree of 29 May 2013, and accordingly only after review and approval of an independent ethics committee. Furthermore, these studies were conducted in an AAALAC-accredited animal facility.

### 2.2 Culture of rat primary neurons *in Vitro*

Experiments were conducted using rodent cortical cells (Mundy and Freudenrich, 2000; Hogberg and Bal-Price, 2011). Primary neurons were freshly dissociated from embryonic E18-19 rat cortices as previously described (Valdivia et al., 2014; Kreir et al., 2018a) and seeded in 48-well MEA plates (BioCircuit MEA plate, cat. Nos M768-BIO-48, Axion Biosystems). One day before plating the cells, each 48-well MEA plate was pre-coated with a polyethyleneimine (PEI) (0.1%) solution (Sigma, cat. Nos 03,880), washed four times with sterile distilled water, and then allowed to dry overnight. On the day of plating, Laminin (20 μg/mL) (Sigma cat. Nos L2020) was added to each 48-well plate, which was then incubated for 1 h at 37°C. Subsequently, both types of neurons were cultured at 37°C, 5% CO_2_, 95% air atmosphere, in a neurobasal medium (Thermofisher, cat. Nos 21,103-049) supplemented with 0.5 mM L-glutamine (Thermofisher, cat No 25030149) and 2% B27 (Thermofisher, cat No 17504044). At DIV28, spontaneous neuronal activity obtained for 30 min in culture solution was defined as the baseline. Compounds were added at a single concentration per well (n = 7 or eight per concentration) and plates were kept in the incubator (37 °C, 5% CO2 and 95% O2 atmosphere) for 60 min before being recorded for 30 min.

### 2.3 Data analysis of rat primary neurons *in Vitro*

Data analysis was captured using AxIs suite software (Axion Biosystems Inc., version 3.6.2) and further analyzed using GraphPad Prism (version 9.00; GraphPad Software Inc., San Diego, CA). Active electrodes out of the total 16 electrodes per well were defined as an electrode having an average of more than six spikes per minute (0.1 Hz) (Wallace et al., 2015). An active well should have more than 40% active electrodes. All wells below this threshold were discarded upon these quality criteria. For the downstream statistical analysis, the data on each MEA parameter was first expressed as the ratio of exposed wells (percentage change between baseline and the treatment) and then normalized to the average of the control within the same plate. i.e., Baseline corrected ratios of *n= 7 or eight* wells were averaged per condition. Each well of the MEA served as its own control, and the changes in the electrical activity elicited by the treatments were expressed as a percentage of that control activity and normalized to the wells treated with the vehicle control dimethyl sulfoxide (DMSO). The final concentration of DMSO added to each well was 0.1% (1 μL/mL), which did not alter the pH or the ionic concentration of the medium. Differences were determined using the non-parametric Wilcoxon Mann Whitney test; *p-values* below 0.05 were considered significant. Data are expressed as means ± S.E.M.

### 2.4 Selection of MEA parameters

The selection of key parameters is crucial to determining the hazard of compounds. In our previous work (Kreir et al., 2018a), we used 12 parameters to determine the effects of compounds in rat cortical neurons. These parameters reflected relevant changes related to pharmacological effects and preferentially differentiated the severity and direction of the effect, excitatory or inhibitory. Excitatory compounds present a higher risk of acute seizures whilst inhibitory compounds can reduce the threshold for seizures.

To be unbiased of the type of parameters used and to match our type of neuronal culture (strain of rats, duration of culture, media, and supplement used) we used a statistical model, LASSO regression (Tibshirani and Suo, 2016; Ahmed et al., 2022), to analyze all the parameters extracted from the MEA recordings pre- and post-compound additions. This model was used to select the most important change parameters among 43 parameters of the MEA recordings pre- and post-positive and negative controls. LASSO is a linear regression that uses shrinkage to produce simple models with fewer parameters by excluding highly correlated parameters. The linear model is defined as Y=Xβ+ε

Where X is a matrix of highly collinear covariates, β is a coefficient vector, Y is a response vector (compound group: positive-, negative control or vehicle), and ε a stochastic component. LASSO performs variable selection and regularization to increase the prediction accuracy of the above linear regression. The performance of LASSO is evaluated using the balanced error rate (BER: the proportion of subjects for whom we make the wrong classification: BER = 0.5*(FP/(TN + FP) + FN/(FN + TP)), (where FP: false positive, TN: true negative, FN: false negative and TP: true positive) the positive predictive value (PPV), PPV = TP/(TP + FP), negative predictive value (NPV), NPV = TN/(TN + FN), and the Cohen’s Kappa (κ) which is a simple percent agreement (κ = 2*(TP*TN–FN*FP)/(TP + FP)*(FP + TN)+(TP + FN)*(FN + TN)). Cohen’s Kappa (κ) considers the possibility of the agreement occurring by chance. From the data, two sets were used, a training set and a testing set. The training set is used to fit the linear model and the test set is used to evaluate the performance of the prediction. This was repeated 100 times to establish a rank of parameters based on the frequency of being selected.

### 2.5 Defining the cut-off for the scoring system

Thereafter, based on the effects of vehicles and positive controls, we determined the cut-off points between the different effect zones according to the key parameters selected as described in the previous subsection. The zones were defined as follows. The “no effect” zone reflects parameter changes that are most likely within vehicle variability. The “mild” and “strong” zones are differentiated bidirectionally showing mild and strong effects.

The respective hazard class was determined by a cumulative scoring of individual parameters using a scoring matrix, which was based on the following three fundamental components: (1) selection of the six key relevant parameters, (2) defining the cutoffs for the level of effect, and (3) defining weighted points per parameter for the level of hazard potential. The scoring matrix concluded with the addition of a weighted point algorithm to discriminate distinct degrees of neuronal hazard. Statistical tolerance intervals (TIs) (Supplementary Table S2) are used to obtain the cut-off values (Kopljar et al., 2018). The weighted points were defined on the importance of a parameter (and the direction of effect), as well as the identification of an expected hazard for different pharmacological modes of action. The overall hazard score is derived from the sum of all weighted parameters, which could be converted into a specific hazard label. The weighted points and total score range associated with the various hazard labels were optimized using an iterative process.

### 2.6 Prediction model using Random Forest

A Random Forest classification method was used to model all parameters and estimate the probability that a compound is inhibitory, excitatory, or DMSO-like (Brown et al., 2016; Cabrera-Garcia et al., 2021). Random Forest is a commonly used machine learning, which combines the output of multiple decision trees, here the parameters, to reach a single result. It handles both classification and regression problems.

### 2.7 Analysis of sensitivity, specificity, and predictivity values

We analyzed the acute neuro effects of 113 reference drugs with known degrees of risk in humans using the hazard scoring system. We compared the known seizure risk of these reference drugs based on our scoring system to the potential for clinical outcomes (link). Based on the numbers of true positives (TP), true negatives (TN), false positives (FP), and false negatives (FN), we calculated sensitivity (TP/(TP + FN)), specificity (TN/(TN + FP)) and balanced accuracy ((sensitivity + specificity)/2.

### 2.8 Validation of the translational value *in Vitro* into *in Vivo*

### 2.9 Mouse behavioral studies and assessment of acute tolerability

Adult C57BL/6J intracerebroventricular injection:

Phenotypic monitoring with mice was performed where risks were associated with phenotypic observations (convulsion, ataxia, wash and groom, scratch, loss of righting, back LMA, LMA, and video-tracking). High risk was defined as a convulsive activity in at least 50% of the animals tested; Medium risk was defined as no or low convulsive activity and ataxia in at least 50% of the animals tested and finally low risk was where no convulsion was observed, and ataxia was observed in less than 50% of the animals tested.

Electroencephalogram (EEG) rodent studies and assessment of seizures: Male rats (n = 8) (Sprague-Dawley), 400-800 g, were surgically implanted with Stellar Implantable Transmitter (E−430001-IMP-77, Type PBT-M-C Pressure, Biopotential, Temp; TSE systems USA) and microchipped with RFID (Bio13. Therm.03V1 PLS, Biomark Inc, USA) transponder for measurements of activity and body temperature. EEG was used to confirm that convulsive activity is linked to seizurogenic activity measured on the EEG.

## 3 Results

### 3.1 Introduction of the new neuronal hazard scoring system in rodent primary cortical neuronal cultures using MEA assay

**FIGURE 1:**
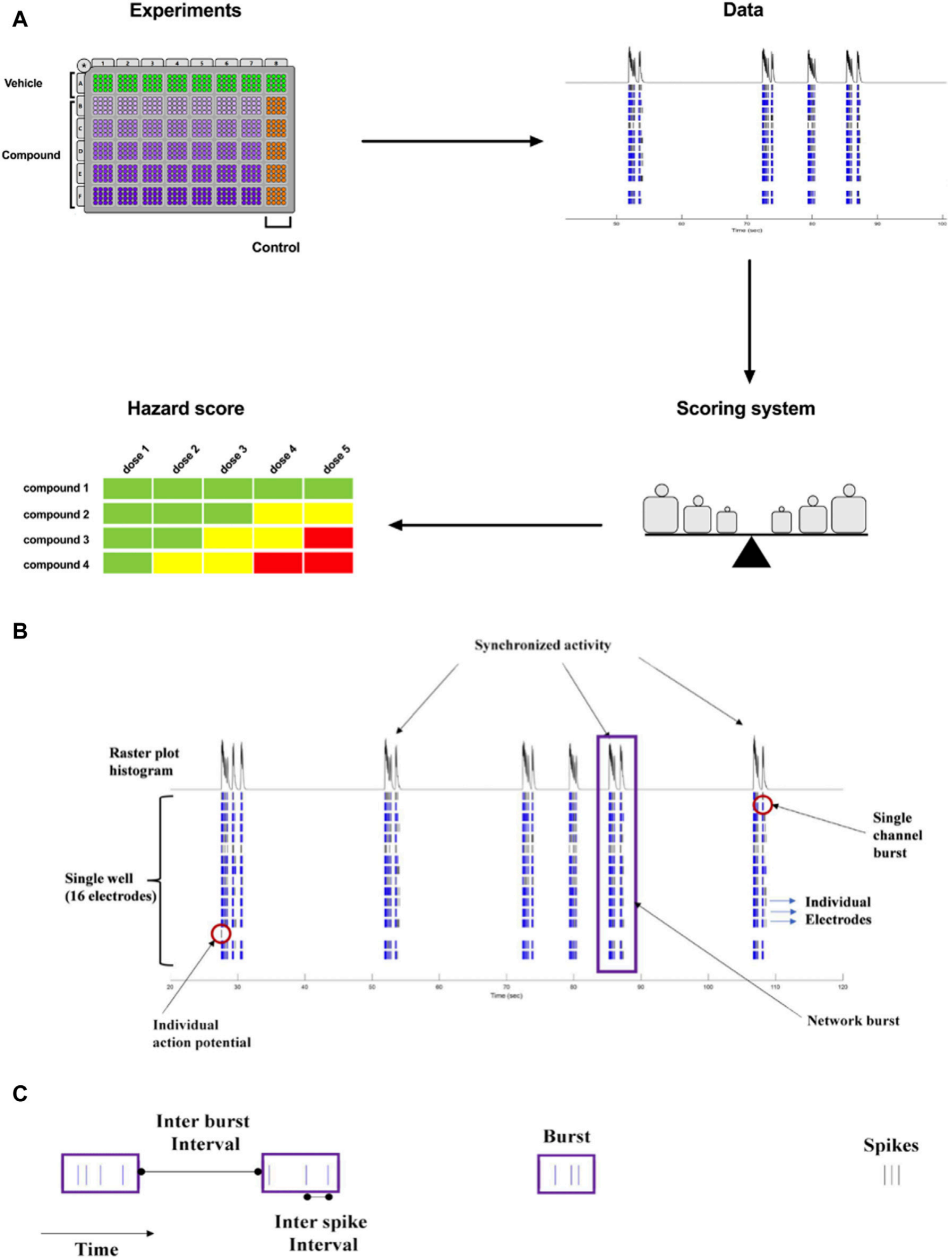
**(A)**. Concept of Workflow on the identification of acute neuronal hazards. The compounds were tested in 48 well plates cultured with primary rat neurons using MEA. Effects (Data changes) in rat primary neurons were analyzed based on several measured parameters, measured in MEA, to have accumulated data points to define the range of scores. This strategy to determine a concentration-dependent hazard and to rank compound candidates is represented schematically. **(B)**. Characteristic recordings of rodent primary cortical neurons. Electrophysiological spiking activity of primary cortical neurons in a single well in an MEA depicted in a time-sequenced raster plot. A vertical bar is drawn each time a neuron fires an action potential (black tick mark), and a burst of spikes is represented in blue on an MEA recording. The main parameters are depicted in **(B)** (16 electrodes) and **(C)** (single electrode).

The goal of our current work was to translate the pharmacological effects of experimental discovery compounds on rodent primary cortical neurons using a phenotypic readout, applying micro-electrode arrays-MEA measurement, to one new hazard score system for new compounds in early drug discovery (Figure 1A).

The MEA assay is a medium-throughput screening tool that is used for the early screening of compounds. In neuronal cultures, the spontaneous electrical activity consists of action potentials (spikes) and patterns of action potential bursts arranged throughout time and space within the network. The MEA recordings provide important information on the activity of the neural network, resulting in high-content data (Figures 1B, C). Many studies have used changes in the mean firing rate as the only metric to observe chemical effects since it is sensitive and can be retrieved rapidly from data sets (Shafer et al., 2008; Novellino et al., 2011; Hondebrink et al., 2016). However, multiple characteristics are required *in vitro* to define and differentiate the drug-induced seizurogenic potential (marked by a partial or total change in the firing activity pattern and network activity) from the excitatory potential (e.g., cognitive enhancer) such as burst duration, the synchronicity of the network activity or the burst and spike organization (e.g., median over mean interspike interval). From the high-content data generated from the MEA, many parameters can be extracted and used for classification (Hammer et al., 2015), however, many parameters are correlated and do not fit into a screening paradigm. Therefore, simplifying the high-content MEA data into six key parameters based on our earlier paper (Kreir et al., 2018a) was performed and validated with statistical modeling.

### 3.2 Selecting key relevant parameters for neuronal hazard identification

The evaluation of the spontaneous firing properties of the rodent primary cortical neurons was then done and selected using the following four key parameters: (1) the weighted mean firing rate corresponding to the number of action potentials recorded; (2) the burst duration representing the neuronal firing within a consecutive string of spikes defined as bursts identified using Poisson Surprise method; (3) the area under cross-correlation corresponding to the degree of association between two variables (here the electrodes where bursts or spikes are present) and can be used to assess the relationship between them; (4) the median over the mean inter-spike interval corresponding to the measurement of spike organization within bursts. Those parameters were also confirmed using a correlation method, where highly correlated parameters were found and allowed to reduce the number of parameters. For this, statistical modeling was used (LASSO regression) to support and establish the most common parameters changed after drug treatment. LASSO is a linear regression that uses shrinkage to produce simple models with fewer parameters (Supplementary Figure S2). The parameters selected in the scoring system were shown to appear at high frequency in the LASSO model. Therefore, not all parameters were selected for the scoring system and only four of the LASSO model exercises were finally selected based on iteration. Based on a scenario where we used as group DMSO and negative controls against inhibitory compounds and excitatory compounds, and timepoint and dose are not penalized, we found 15 parameters for the inhibitory trend (with ≥50% frequency) and 7 parameters (with ≥50% frequency) for the excitatory trend (Supplementary Figure S2). Inhibitory and excitatory compounds shared similar parameters reducing the numbers to 13 parameters. A stepwise regression was then used to further see if all parameters were needed. Stepwise regression (or stepwise selection) consists of iteratively adding and removing predictors, in the predictive model, to find the subset of variables in the data set resulting in the best-performing model, which is a model that lowers prediction error. The BER was used to see how many parameters are needed to detect properly the positive controls *versus* vehicle and negative controls (Supplementary Figure S3). From there, we selected four parameters (weighted mean firing rate, burst duration, area under cross-correlation, and the median over mean interspike interval) that were highly recommended by the LASSO regression result. The reduction of parameters did have slight significant changes on the BER distribution, and we further decided to keep two additional parameters: cessation of neuronal firing (FS) and cessation of the neuronal network (NS) from extra observations (when increase of firing and burst occur outside network activity until complete asynchronous activity between electrode are observed), because these two parameters measured in MEA, are pharmacologically and physiologically relevant, but not specifically reported in the outcome of standard MEA measurements. For example, compounds at high concentrations can have a strong inhibitory or toxic effect on neuronal electrophysiology that can lead to a stop of action potentials or disturb the network burst formation without impacting consequently the firing rate. Although there would be no information on the effects of the primary parameters, those parameters indicate a relevant pharmacological response in primary neuronal cultures.

## 4 Scoring matrix for the new hazard scoring system: Defining cutoffs and weighted points

**FIGURE 2:**
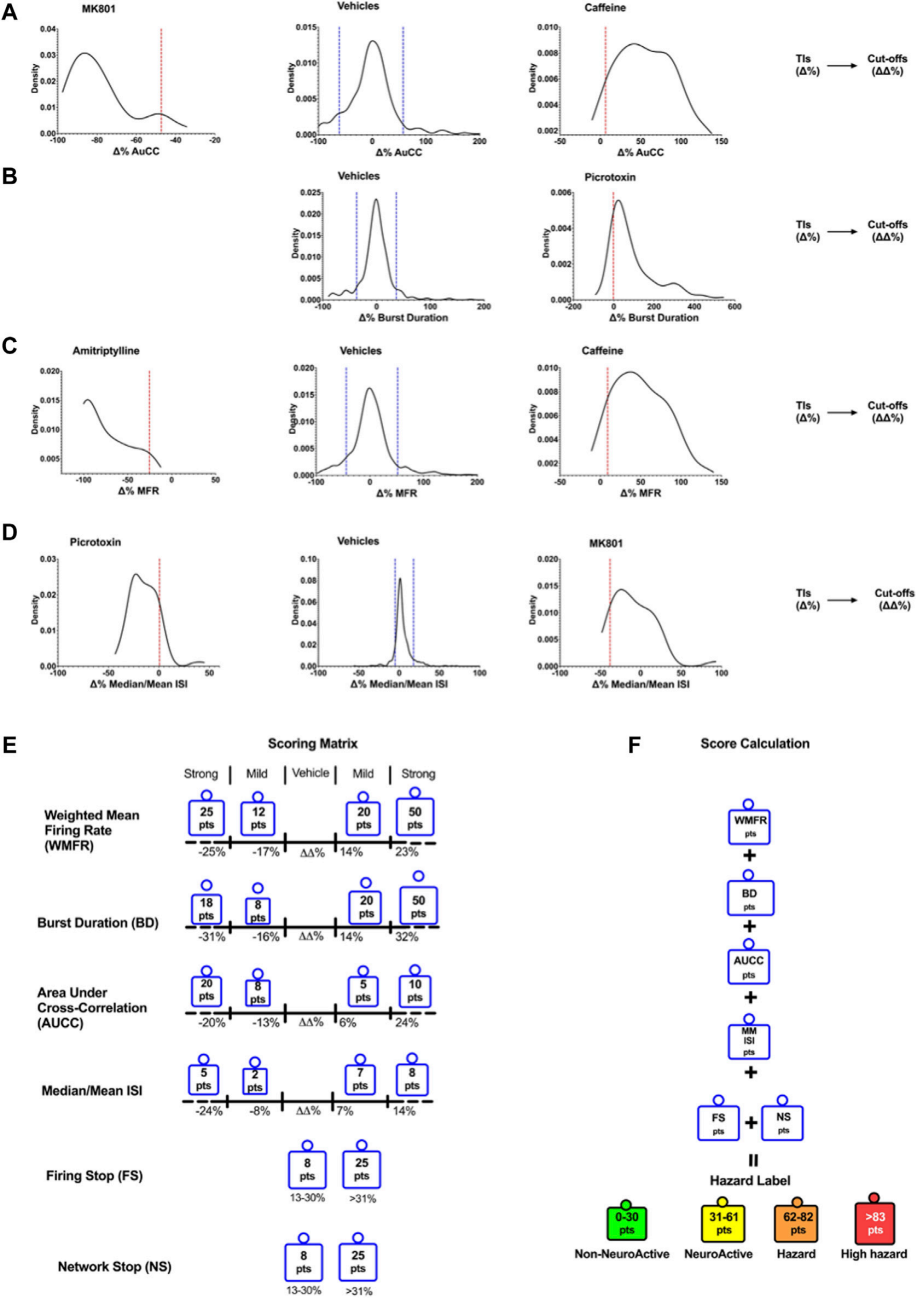
Density plots show the TIs for vehicles and positive controls calculated for the six selected key parameters. Determination of Cutoffs using TIs to develop the scoring matrix. Examples of the TIs of compounds on AuCC **(A)**, the burst duration **(B)**, the weighted mean firing rate **(C)**, and the median/mean Inter-Spike Interval (ISI) **(D)**. The blue line corresponds to the upper and lower bound Tis for the vehicle, and the red line corresponds to the upper and lower bound Tis for the compounds. **(E)** The scoring matrix represents a point card where for each parameter a weighted score is given depending on the size and direction of the ΔΔ% effect. **(F)** Calculation of hazard scorings is done through a sum of points across all six parameters.

Figure 2 shows the cut-off points between the different effect zones according to the six key parameters.

The outcome of neuronal scoring for the tested compound was classified into the following four categories (color labels): “non-neuroactive” (green), “Neuroactive (yellow), “Hazard” (orange), and “high hazard” (red). Non-neuroactive labeling indicates compound effects within the vehicle variability (Figure 2). Non-neuroactive (Green): is defined without significant changes in 6-key parameters of the MEA and within the vehicle and negative control variability; Neuroactive (Yellow): small but significant effects on 6-key parameters (slightly and clearly above the vehicle variability) without risk; Hazard (Organ): Mild effects on the six key parameters with limited risk. High hazard (Red): Shows strong changes on six key parameters and suggests a great concern that could lead to CNS adverse effects.

**FIGURE 3:**
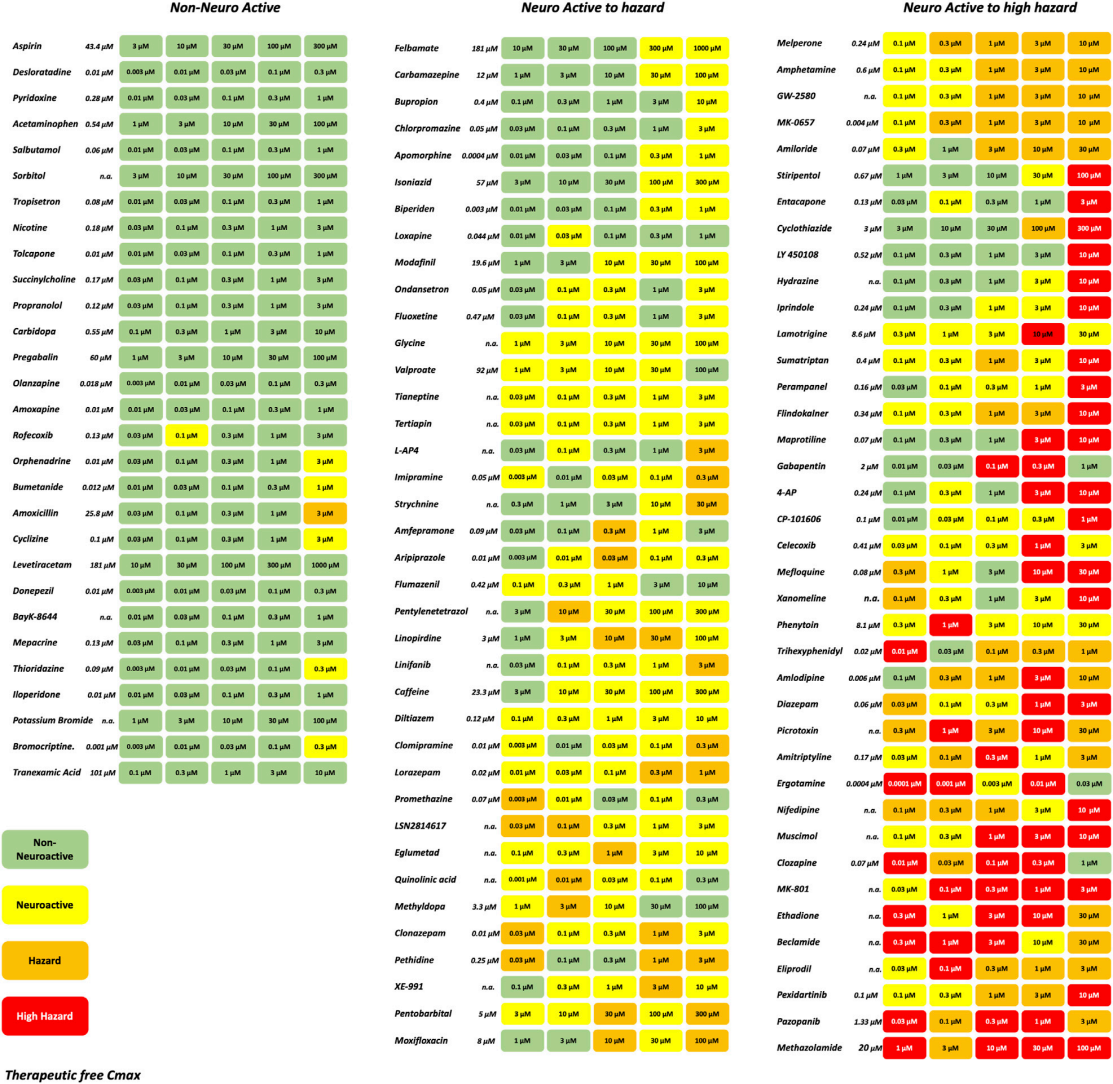
Effect of representative 106 reference drugs on the rat cortical neurons measured on the MEAs using the hazard score system defined by the electrophysiological changes per concentration. Concentrations were selected based on the therapeutic-free Cmax (shown in italics). n. a., not available. Green: no risk (non-neuronal active), yellow: neuronal active; Orange: hazard, and Red: high hazard.

A set of in total 113 reference compounds with known pharmacological or seizurogenic outcomes (drug-induced seizure in man or preclinical species, neuroactive and non-neuroactive drugs) was used to develop and optimize different parts of the scoring system (Figure 3). Concentrations were selected to cover therapeutic-free peak plasma concentration (C_max_), when applicable. Negative control drugs were chosen for their low number of reports or studies of seizure liabilities in humans or preclinical species and are expected to be identified as non-neuroactive for concentrations several folds the free Cmax (Supplementary Table S1). The results of the hazard scoring of 106 reference drugs at different concentrations, relative to their fCmax, on rat primary neurons are presented in Figure 3. The outcome of the acute hazard scorings predicted well and differentiated non-neuronal active drugs (safe), from neuronal active or from drugs with clinical seizure risks.

### 4.1 Analysis of sensitivity, specificity, and balanced accuracy

Classification of drugs inducing seizures is complicated and depends on the dose taken. The higher the dosage, the higher the risk of observing convulsion in man. Many drugs where convulsions were reported to occur in overdose conditions and not in the therapeutic dose range. To be able to classify our reference compounds, we used several sources of information: WHO adverse drug reactions (ADR) VigiBase (Kumlien and Lundberg, 2010), SIDER side effect resource (Campillos et al., 2008; Kuhn et al., 2010), drug information provided by Elsevier, and the Food and Drug Administration (FDA) approved prescribing information (National library of medicine, Daylimed). From this information, we established the degree of risk for the drugs from low seizure risk to high seizure risk (Supplementary Table S1). Antiepileptic drugs and anesthetic drugs acting in the CNS were included in either the neuroactive or high-risk category. Many anticonvulsant drugs show hazards such as phenytoin, diazepam, perampanel, or clonazepam are considered anticonvulsants with the potential risk of reducing the threshold for seizures at toxic concentrations (overdose) (Gayatri and Livingston, 2006).

**FIGURE 4:**
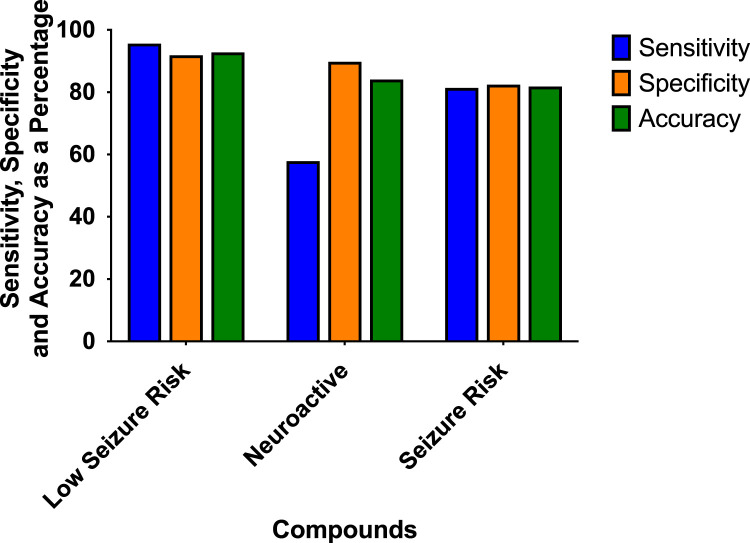
Sensitivity, specificity, and accuracy were derived from the true positive, false negative, false positive, and true-negative classification of the 113 reference compounds on the outcome of the hazard scoring system.

The prediction values for the scoring hazard identification are based on 113 reference drugs with known clinical outcomes of seizures as shown in Figure 4. It shows high sensitivity (95.65%), high specificity (91.89%), and high accuracy (92.78%). Only one compound, amiloride, out of 113 compounds was wrongly classified, false positive, as having a high seizure risk, whereas it has a low seizure potential in men. For the neuroactive compounds, the outcome, as we expected, was identified with low sensitivity (57.89%), but with a high specificity of 89.78% and an accuracy of 84.11%. The risk class showed a sensitivity of 81.43%, a specificity of 82.5%, and an accuracy of 81.82%.

### 4.2 Using statistical Random Forest to further classify the activity of compounds

**FIGURE 5:**
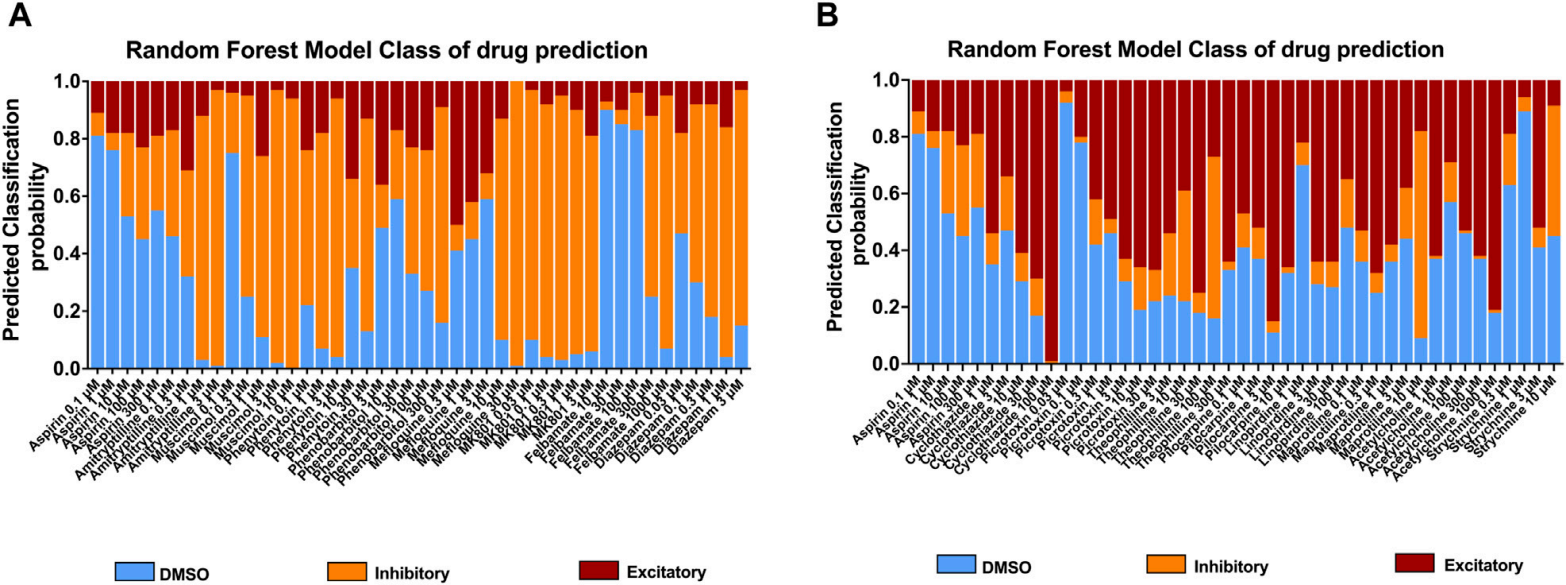
Classification of 18 compounds into no effect (DMSO), **(A)** inhibitory, and **(B)** excitatory drugs by using Random Forest prediction. The results show the probability that the compounds will be classified into their categories at each concentration, allowing an addition to the hazard identification score system.

To further understand the effects of the compounds, we used statistical modeling using Random Forest. This method allows us to classify compounds under a different category such as inhibitory effect or excitatory effect and thus discriminate the drugs inducing seizures via excitatory from the drugs that have the potential to lower the threshold for seizures or neuroactive drugs that decrease the activity of the neurons, e.g., sedatives. The outcome of this statistical modeling is shown in Figure 5 and supports the hazard identification score system on MEA measurements.

### 4.3 Assessment of newly synthetized compounds from the hazard scoring system and translational value from *in vitro* hazard scoring to *in vivo* in mice

**FIGURE 6:**
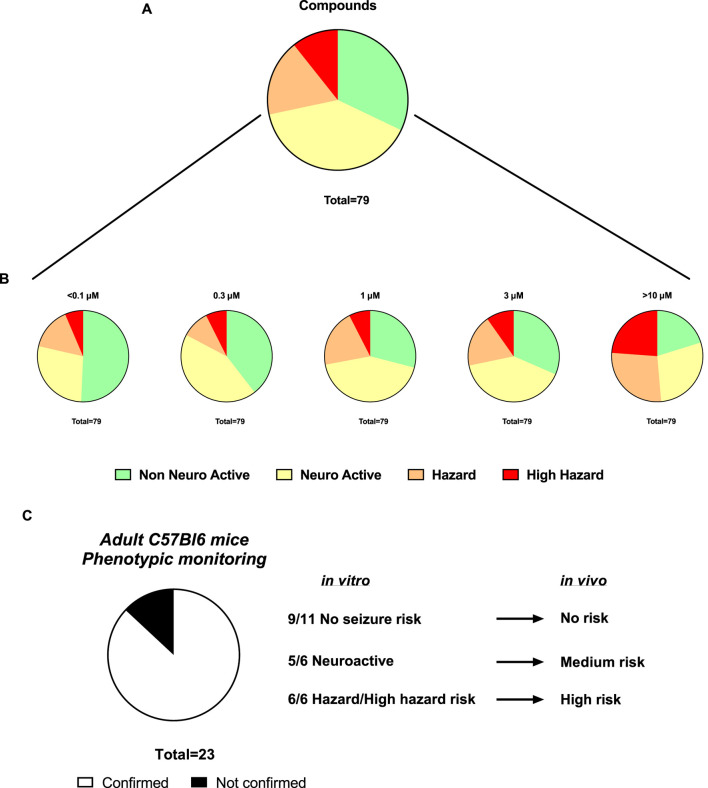
Assessment of the Hazard scoring of newly synthetized compounds on rat primary neurons measured by MEAs. **(A)**. Pie charts showing the total distribution of different hazard levels for compounds regardless of concentrations (n = 79). **(B)**. Pie charts showing the concentration-dependent distribution of different hazard scorings for compounds (n = 101). **(C)**. Translational confirmation of the MEA-derived neuronal hazard scoring of 23 antisense oligonucleotides (ASOs) that were also evaluated in a phenotypic monitoring assay *in vivo* using adult C57Bl6 mice.

Sequentially, we investigated 79 newly synthetized compounds, 70% of which had activity on one or more neurological targets. The compounds were tested in a wide concentration range (0.1–10 µM). As expected, most of the neurological target compounds have effects on the Hazard score system as expected as the target is present in the brain (Figure 6): Hazard evaluation identified that most compounds were classified within the “no hazard -non-neuronal active” or “neuronal active group at concentrations less than 10 μM, with a portion showing risk at the highest concentration of 10 µM (Figure 6B). Compounds (35.15%) were found not to be a hazard (non-neuronal active *in vitro*) and 35.34% to be neuroactive over the entire concentration range (Figure 6A). Additionally, for compounds identified as hazards, the levels increased in a concentration-dependent manner (Figure 6B). At 1 µM and higher, more than 18.45% of the compounds showed some effects associated with a certain level of hazard with the “high hazard” mainly at 10 µM (11.07%).

Subsequently, the preclinical translational predictability of scored compounds that have been also tested in *in vivo* models was evaluated. The compounds tested in both *in vitro* and *in vivo*, are from the antisense oligonucleotides (ASOs) class hitting a variety of targets and thus have varying sequences. Figure 6C demonstrated a good translational value of 23 ASOs from the hazard scoring *in vitro*: the confirmation rate from data *in vitro* to the adult mice was 87%. In two cases, low risk *in vivo* showed neuroactive effects *in vitro* and one case of medium risk *in vivo* showed high seizure risk *in vitro*.

**FIGURE 7:**
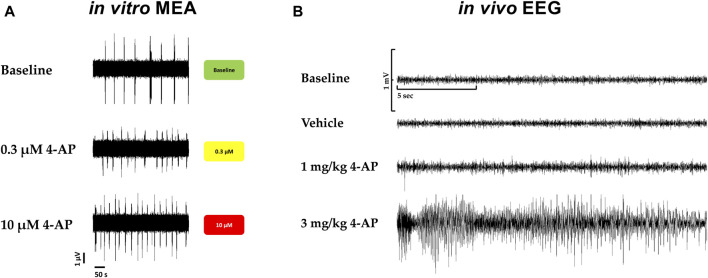
Translational confirmation of the MEA-derived neuronal hazard scoring of a reference compound (4-AP) that was also evaluated in a freely moving rat with EEG recordings. **(A)**. Effects of 4-AP on the MEA *in vitro* with the hazard score for 0.3 and 10 µM 4-AP. **(B)**. EEG recordings in a freely moving rat at baseline and vehicle and in the presence of 4-AP at different doses, 1 and 3 mg/kg.

Furthermore*, in vivo,* convulsions in rats associated with behavioral seizures, induced by 4-aminopyridine, a selective potassium channel blocker (Kv1) known as being a potent convulsant agent and used to generate seizures in animal models for the evaluation of antiseizure agents, were compared with the hazard-scoring system *in vitro* (Figure 7). Figure 7 shows the electroencephalogram (EEG) with 4-aminopyridine (Figure 7B) and the presence of behavioral manifestations of seizure activity in a freely moving rat in comparison with the hazard score system and raw traces obtained from the MEA recordings (Figure 7A): 4-AP significantly increased EEG spikes (1 mg/kg, *iv*) and induced convulsions and seizures at 3 mg/kg, *iv*, with the appearance off abnormal discharges in bursts (see example in Figure 7B) while 4-AP also largely increased the firing rates similarly *in vitro* with MEA recordings.

## 5 Discussion

Seizure liabilities are serious life-threatening adverse effects and are often used as a showstopper for the progression of a compound for further development in the clinic. Two industry surveys indicated that seizures and tremors represented most of the CNS problems encountered in preclinical studies (Nagayama, 2015; Authier et al., 2016). In the present study, we developed and introduced a new hazard-scoring approach using rodent primary cortical neurons cultured on MEA to aid in the selection of compounds devoid of potential drug-induced seizure risk. Our data show that the MEA assay can identify effects on the CNS by various compounds with different targets as reported by previous studies (Valdivia et al., 2014; Vassallo et al., 2017; Bradley et al., 2018; Strickland et al., 2018; Kosnik et al., 2020; Tukker et al., 2020; Saavedra et al., 2021). We applied the same principles of the previous hazard score system used on another organ cell line (Kopljar et al., 2018) to rat primary neurons, using statistical TIs and cut-off values for the key parameters extracted from MEA recordings, based on DMSO controls, positive controls, and validation set of 113 known reference drugs that included both positive and negative controls for various parameters. Statistical analysis (TIs) on a large data set of vehicles and control drugs helped to develop a detailed scoring system with differentiation of size and direction of effect for each parameter. We showed that the hazard scoring system together with the Random Forest analysis allowed us to simplify the interpretation of drug-induced effects on multiple parameters measured (weighted mean firing rate (WMFR), burst duration, AuCC, median/mean ISI, network cessation, and firing cessation) from the neuronal cultures and allowed to differentiate various pharmacological classes of drugs.

**FIGURE 8:**
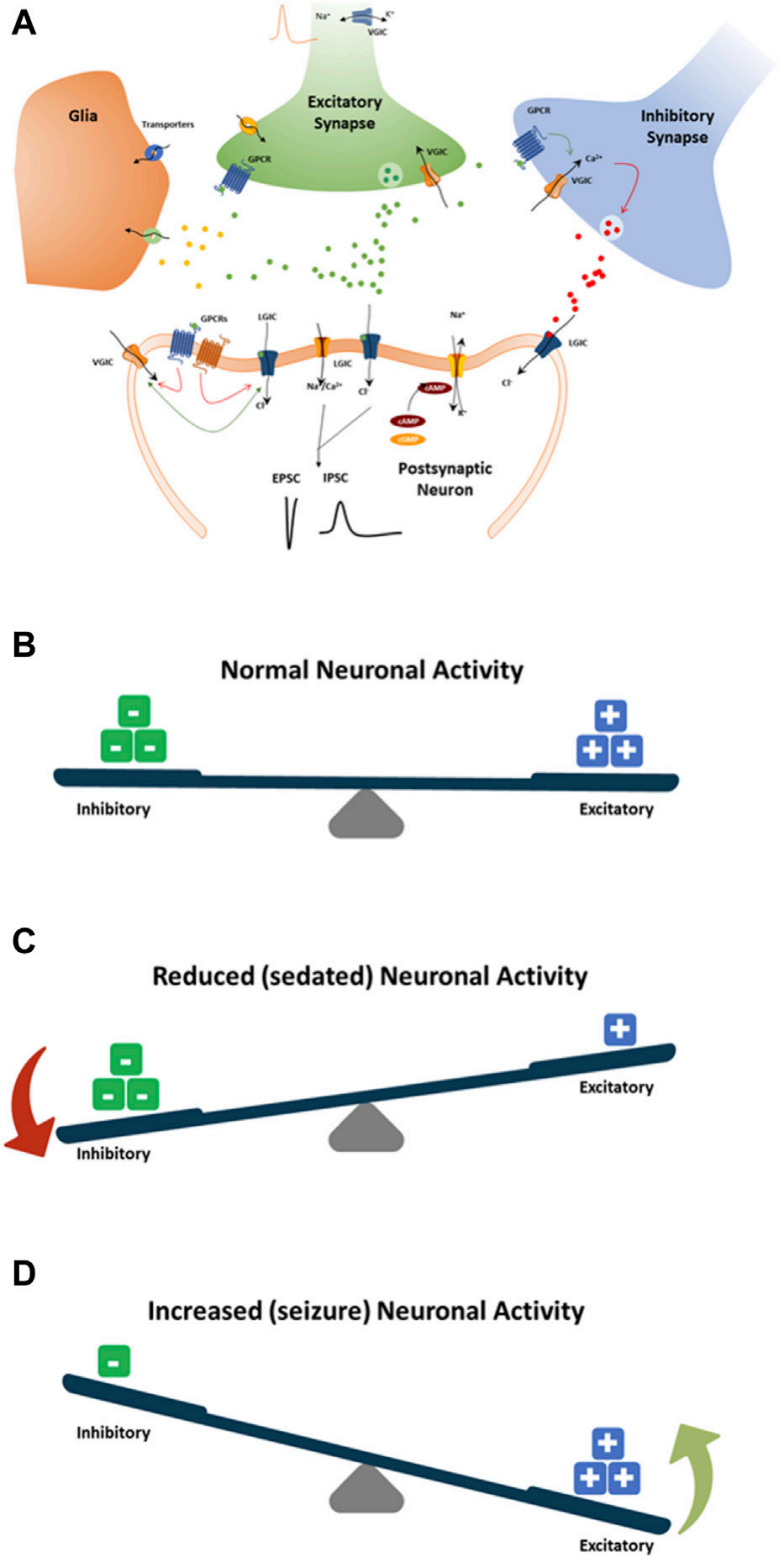
Schematic showing standard paradigm for understanding the balance between excitation (+) and inhibition (−) in drug-induced seizures **(A)**. **(B)** The normal balance between excitatory and inhibitory neuronal activity, receptor and ion channel function, neurotransmitters, and pathways. **(C)** Modulation of neuronal receptors or ion channels by drugs that decrease neuronal activity by increasing inhibitory neuronal activity or decreasing excitatory neuronal activity. **(D)** Modulation of neuronal receptors or ion channels by drugs that increase neuronal activity by increasing the excitatory neuronal activity or decreasing the inhibitory neuronal activity. Both **(C, D)** will result in an imbalance toward possible seizure occurrence.

Mechanisms by which drugs induce seizures are complex and are not always understood. However, an unbalance of electrophysiological activities in the neuronal system could cause excitatory or inhibitory effects on the CNS (Figure 8). Therefore, we speculate that disruption (i.e., modulations of neuronal receptors and ion channels) in the synaptic balance between excitation and inhibition in monolayer rat primary neurons could lead to potential mechanisms of drug-induced seizure (Figure 8). The most prominent mechanism causing an unbalance is often reported to be via modulation of the gamma-aminobutyric acid (GABA) receptors (i.e., inhibitors, agonists … ), such as pentylenetetrazol, bicuculline and benzodiazepines. Enhancing the activity of the excitatory neurotransmitter glutamate by modulating the metabotropic glutamate receptors (mGluR) or the ionotropic glutamate (iGlu) receptor, N-methyl-D-aspartate (NMDA) receptors, permeable α-amino-3-hydroxy-5-methyl-4-isoxazole propionic acid receptor (AMPA receptor), Kainate receptors and delta receptor family (Wang and Gruenstein, 1997; Dravid and Murray, 2004). Additionally, modulation of voltage-gated sodium channels, potassium channels, and Ca^2+^ channels, the ATP-gated P2X receptor cation channel family (P2X receptor), the transient receptor potential (TRP) superfamily of cation channels, and acid-sensing ion channels are also known to be associated with neuronal processes. Other less known mechanisms are the increased dopamine release or blocking dopamine reuptake, leading to hyperactivity in the dopaminergic system that can lead to seizures. Drug-inducing mitochondria dysfunction has also been reported as a potential increased risk of seizures (e.g., valproic acid, metronidazole) (Zsurka and Kunz, 2015).

To determine the risk class of marketed compounds, we used different sources from the VigiBase^®^ (WHO) (Kumlien and Lundberg, 2010), the SIDER side effect resource (Campillos et al., 2008; Kuhn et al., 2010), the FDA Adverse Events Reporting System (FAERS), and drug information provided by Elsevier and the FDA-approved prescribing information (National Library of Medicine, Daylimed) (Supplementary Table S1). This allowed us to establish some evidence of a certain degree of potential convulsive/seizurogenic risk. The hazard identification system could detect seizure risk for most of the high seizure hazard drugs within the 30-fold free C_max_ range. A few high seizure-risk compounds were not correctly classified such as amoxapine, well known to induce seizure in men, imipramine, donepezil, and tranexamic acid. Both amoxapine and imipramine share the same mechanism of action (reuptake inhibitor of norepinephrine and serotonin) which is difficult to identify in rodent cortical neurons measured on the MEA, as we reported earlier (Kreir et al., 2018a). Donepezil, an acetylcholinesterase inhibitor, did not induce any changes *in vitro* up to 30-fold its free C_max_. Acetylcholinesterase inhibitors are known to have the potential to lower the threshold for seizure and provoke seizures (Fisher et al., 2001; Kumlien and Lundberg, 2010). Lowering the seizure threshold *in vitro* can be challenging if no obvious inhibitory effects are observed, thus limiting the detection of such compounds. On the other hand, tranexamic acid was only used at its therapeutic dose due to the high free C_max_ concentration (>100 µM) in man and was safe up to 1.5-fold its free C_max_.

Compounds classified as low risk were identified as neuroactive within the 30-fold free C_max_ range. However, many low-risk compounds were classified as either seizure or high seizure hazards and a few as non-neuroactive, e.g., nicotine most likely due to its transient effect as described by Hondebrink et al. (Hondebrink et al., 2016). When looking at the class of compounds from the low-risk group in man, whilst classified in our hazard system as high hazard, four out of six compounds were anticonvulsants/anxiolytic (perampanel, phenytoin, diazepam, beclamide), one was a non-steroidal anti-inflammatory drug (NSAID) compound (celecoxib) and the other was an anti-hypertension compound (methyldopa). Many anticonvulsant or anxiolytic drugs are difficult to classify as only neuroactive as their effects can be highly inhibitory (e.g., phenytoin, sodium channel blocker) leading to the cessation of firing, thus increasing the weight in the scoring system. Methyldopa is a prodrug that is metabolized into norepinephrine and acts centrally to decrease the adrenergic neuronal outflow from the brain stem. The effect of norepinephrine has been linked to the modulation of seizures as an anticonvulsant as well as a proconvulsant effect, especially on the activation of α2-adrenoreceptors (Szot et al., 2004). This would provide a partial explanation for the hazard score of methyldopa. In the case of celecoxib, its high selective inhibition of cyclooxygenase-2 has been linked to an anticonvulsant effect (Zandieh et al., 2010; Lim et al., 2019), which would classify celecoxib in the same class as anticonvulsant drugs. Out of the 113 reference drugs, one compound, amiloride, with no risk of seizures was detected as high risk (false positive). Amiloride is an inhibitor of the acid-sensing ion channels (ASICs), the Na^+^/H^+^ exchanger (NHE), the Na^+^/Ca^2+^ exchanger (NCX) (Xiong et al., 2008), and the voltage-gated sodium channels (Kleyman and Cragoe, 1988) and is known to be potentially neuroprotective (Durham-Lee et al., 2011). Amiloride has shown an increase of activity (weighted mean firing rate) in the rodent cortical neurons recorded on the MEA, enough to classify the compound as high-risk in the scoring system. This effect was not fully understood and may be a limitation of the *in vitro* system.

Furthermore, the Random Forest analysis, showed how the compounds were behaving independently from the hazard score system. The probability at each concentration to be either inhibitory, excitatory or within vehicle controls was given for each compound. The Random Forest brings additional information about the degree of inhibition or excitation and together with the scoring system explains the electrophysiological balance or unbalance between neuronal inhibition and excitation leading to the potential risk of seizures.

The studies performed with ASOs have also demonstrated a good correlation between the scoring system *in vitro* and the clinical observations *in vivo* in mice. Both *in vitro* and *in vivo* experiments helped us find a correlation between the number of Guanines in the ASOs and the seizurogenic potential. This was also described in the work of Hagedorn (Hagedorn et al., 2022) on the acute neurotoxicity after intracerebroventricular injection into the mouse brain.

The discovery and development of novel compounds is a long and expensive process, and there is a considerable rate of attrition that results, in part, from a safety concern found later in drug development. This assay and the hazard scoring system defined and described in this study can be readily applied to select the best drug candidates based on safety.

### 5.1 Limitation

The hazard scoring system that we developed provides a reliable way to identify concentration-dependent levels of seizure risk for compounds based on MEA measurements in rat primary neurons using six functional parameters. However, despite the great promise of this scoring system for predicting acute neuronal seizure risks, there are still some limitations for rodent primary neurons: 1. This assay does not show direct seizure recordings as measured by EEG in animal models or humans, 2. There is a low expression of certain genes expression (e.g., Htr1a, Htr2a) in the rat primary cortical neurons, therefore, caused by the lack of predictive seizure risk value of antidepressant drugs such as amoxapine or imipramine (Kreir et al., 2018b). 3. Some compounds have adverse effects via their metabolites formed *in vivo* but not *in vitro* which limits the potential detection of such compounds before *in vivo* models are used. Furthermore, there is a lack of a self-regulation system (homeostasis, acid-base balance, … ). 4. This assay still requires the use of animals (rats); therefore, human iPSC-neurons should be explored in future studies to develop the hazard score system in a human cell-based assay.

## 6 Conclusion

This new acute neuronal hazard score system for detecting drug-induced seizure applied to the MEA measurement using rat primary neurons will be useful for the identification and selection of compounds for further investigation, reducing the overall number of animals used as well as decreasing the associated costs.

## Data Availability

The raw data supporting the conclusion of this article will be made available by the authors, without undue reservation.
