# Supplementary figures and images for: Development of a new hazard scoring system in primary neuronal cell cultures for drug-induced acute neuronal toxicity identification in early drug discovery
Source: Front Pharmacol. 2024 May 30;15:1308547. doi: 10.3389/fphar.2024.1308547 (PMC11170107; doi:10.3389/fphar.2024.1308547)

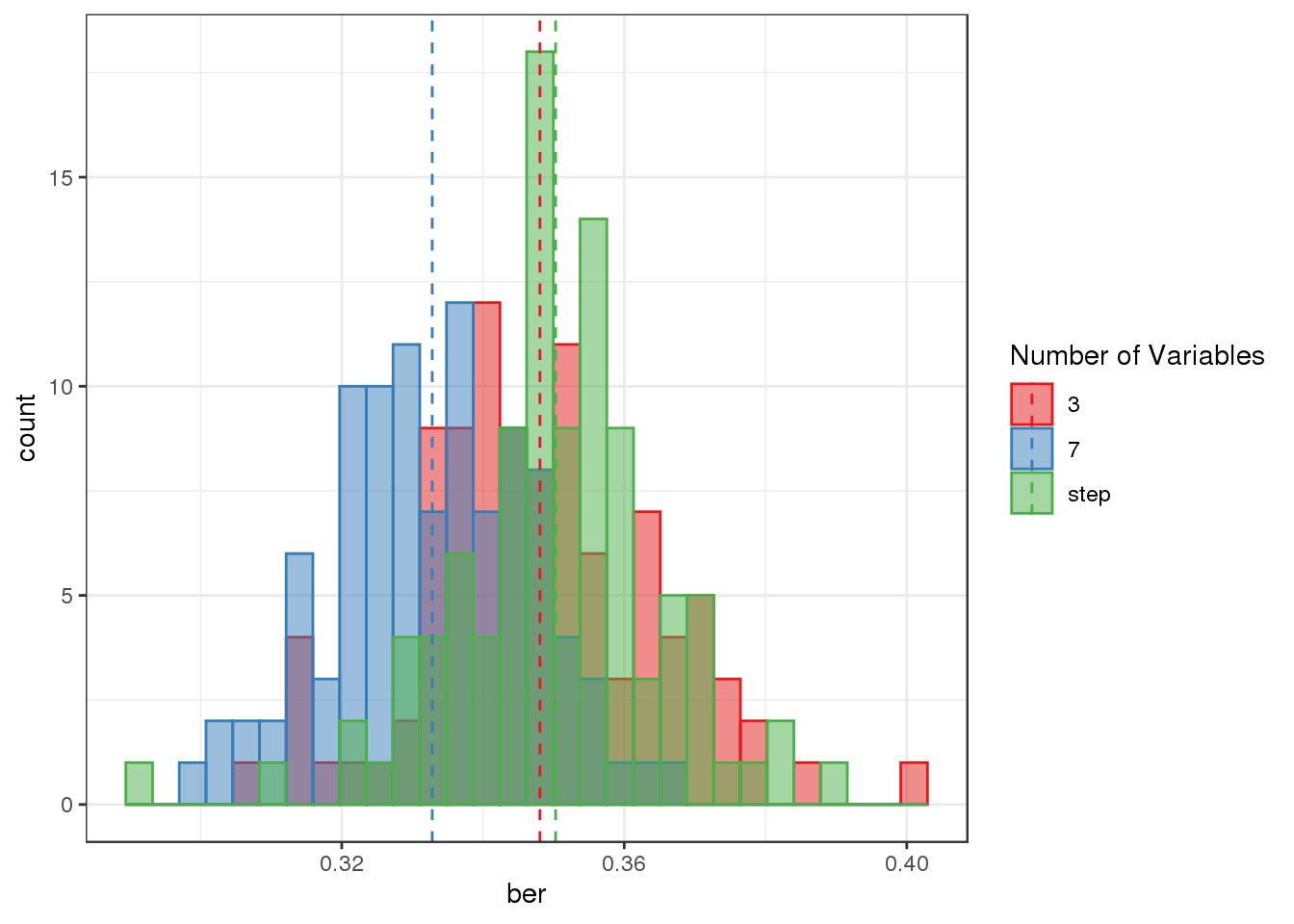

Supplement: Supplementary file 2 [file Image4.PNG]

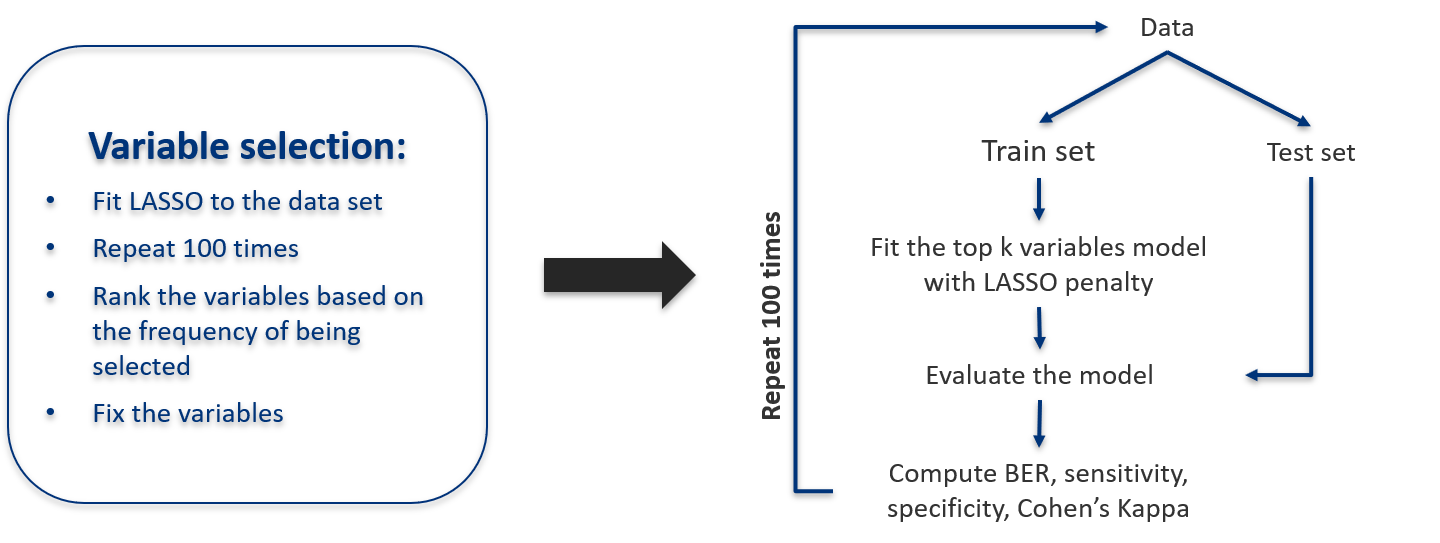

Supplement: Supplementary file 4 [file Image1.PNG]

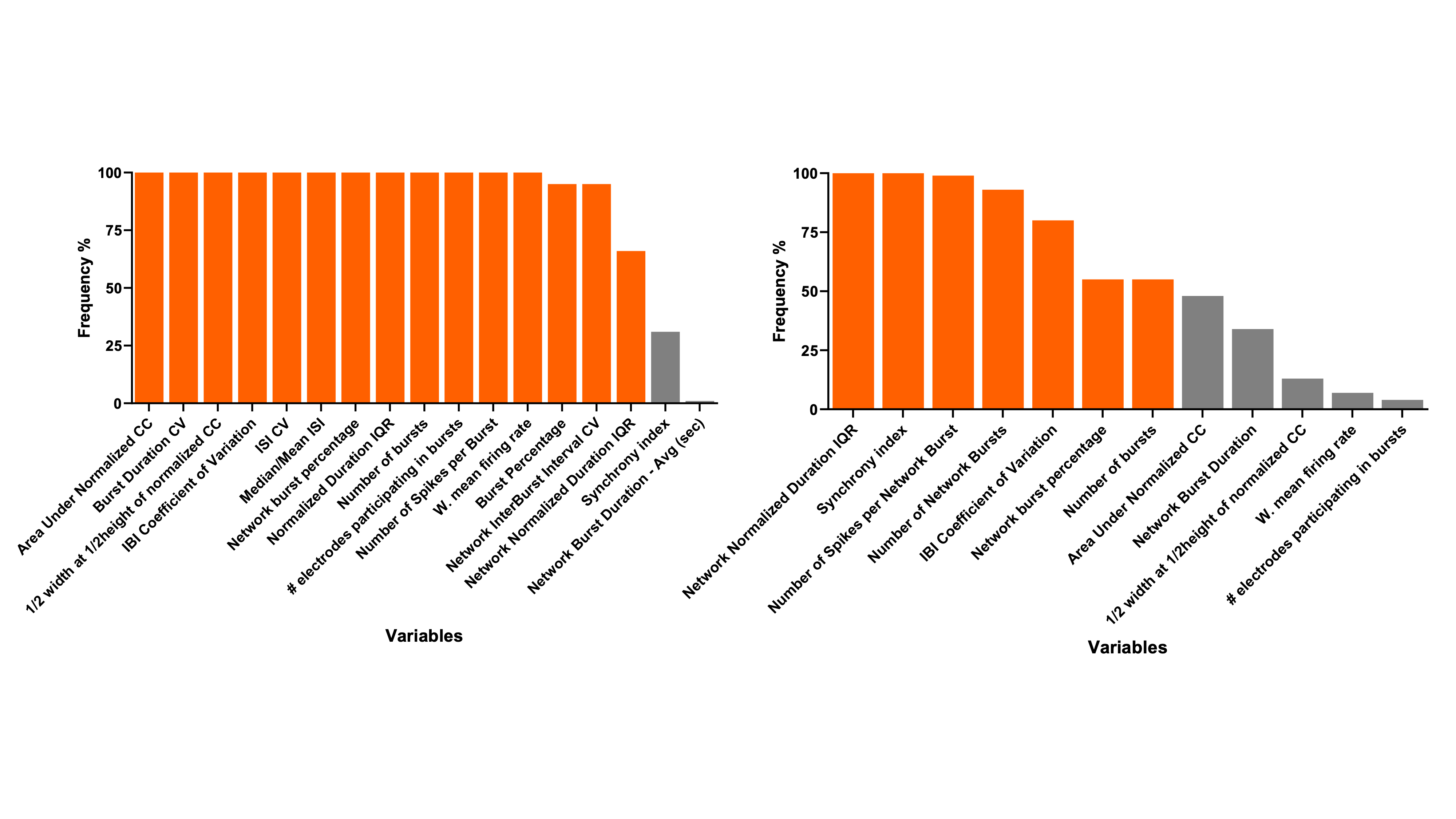

Supplement: Supplementary file 5 [file Image2.TIFF]

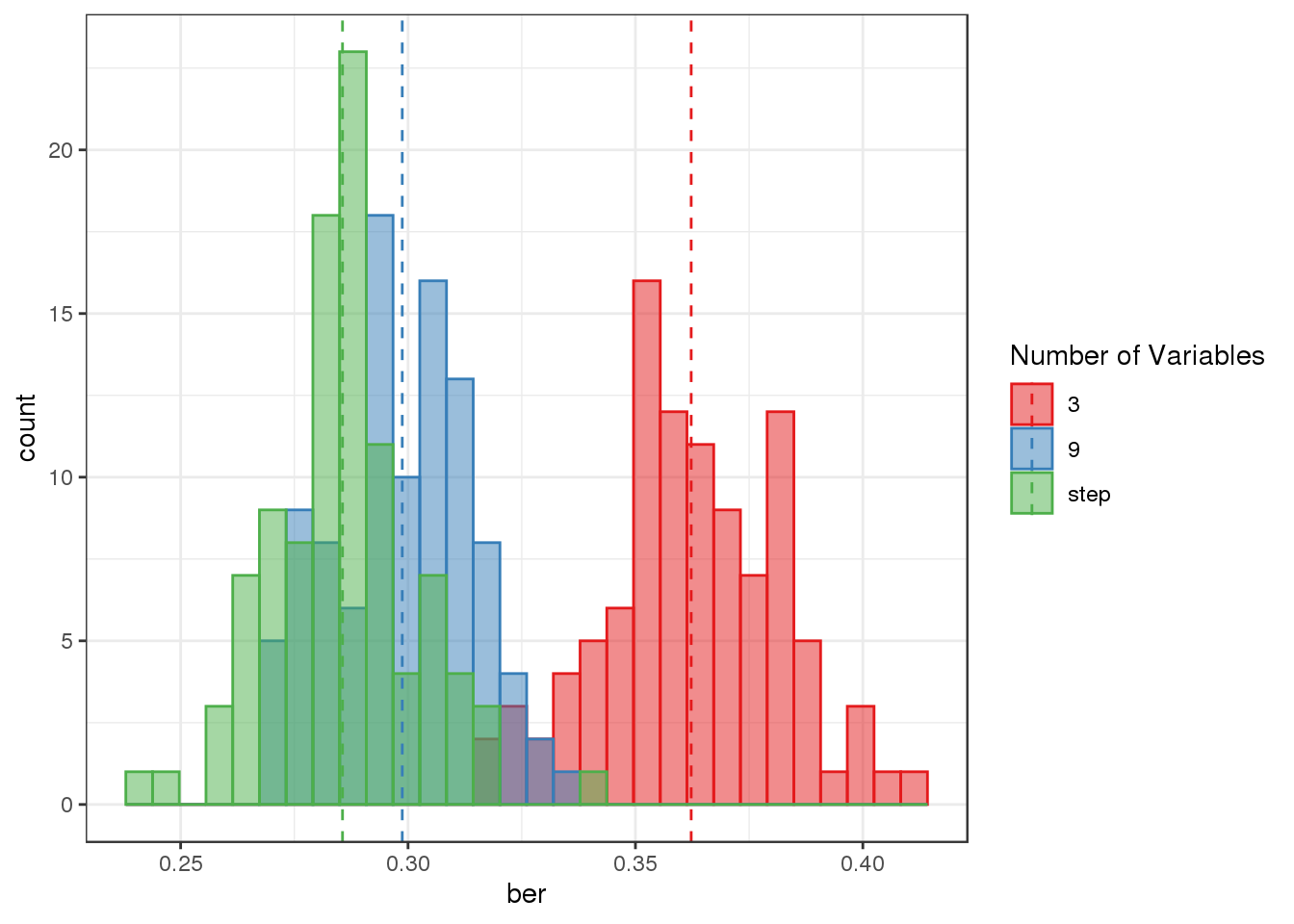

Supplement: Supplementary file 6 [file Image3.PNG]
